# Application of the Stockwell Transform to Electroencephalographic Signal Analysis during Gait Cycle

**DOI:** 10.3389/fnins.2017.00660

**Published:** 2017-11-28

**Authors:** Mario Ortiz, Marisol Rodríguez-Ugarte, Eduardo Iáñez, José M. Azorín

**Affiliations:** Brain-Machine Interface Systems Lab, Miguel Hernández University of Elche, Elche, Spain

**Keywords:** brain-machine interface, EEG analysis, fast fourier transform, gait intention, Hilbert-Huang transform, Stockwell transform

## Abstract

The analysis of electroencephalographic signals in frequency is usually not performed by transforms that can extract the instantaneous characteristics of the signal. However, the non-steady state nature of these low voltage electrical signals makes them suitable for this kind of analysis. In this paper a novel tool based on Stockwell transform is tested, and compared with techniques such as Hilbert-Huang transform and Fast Fourier Transform, for several healthy individuals and patients that suffer from lower limb disability. Methods are compared with the Weighted Discriminator, a recently developed comparison index. The tool developed can improve the rehabilitation process associated with lower limb exoskeletons with the help of a Brain-Machine Interface.

## 1. Introduction

Reduced mobility is a serious handicap for people who have suffered a cerebrovascular accident, brain trauma or encephalitis. Orthesis and prosthesis devices have been developed in last years in order to assist people with severe motor limitations. Although EMG-based interfaces have been used in several applications for controlling these devices (Villarejo Mayor et al., 2017), the use of Brain-Machine Interfaces (BMIs) can be a more suitable option to control a speller or a wheel chair (Li et al., 2014) and especially exoskeletons, since they can improve the neuroplasticity in rehabilitation therapies (Cramer, 2008; Gharabaghi, 2016; Barrios et al., 2017).

The basis of a BMI is to extract the brain waves, normally by electroencephalography (EEG), process and translate them into commands to control a device. The electrical waves obtained are categorized by their frequency components and the location where they are acquired (Rao, 2013). Usually, the following frequency bands are considered: delta (0.1–4 Hz) which is associated with deep sleep (Amzica and Steriade, 1998), theta (4–7 Hz) which is associated with Rapid Eye Movement (REM) sleep and transition from sleep to waking (Cantero et al., 2003), alpha (8–15 Hz) which is associated with relaxed, but awake state with eyes closed (Da Silva, 2010) and beta (15–32 Hz) and gamma (>25 Hz) which are associated with movement and attentive focus (Rao, 2013). Depending on the author, the bands can slightly differ and overlap. Thus, it is hard to establish a precise limit for them. In literature, bands have received another designation; for instance, mu band (8–12 Hz) which is usually related to the event-related synchronization phenomenon (Pfurtscheller and Neuper, 1994). In Cheron et al. (2012), it was demonstrated that some EEG frequency bands (alpha, beta and gamma) are involved in the control of the walking pattern, and that it is possible to extract EEG signals event-related desynchronization/synchronization (ERD/ERS) (Severens et al., 2012; Wagner et al., 2012) from the sensorimotor cortex controlling the contralateral foot placement. This confirmed the study of Gwin et al. (2011) which stated that electrocortical activity is coupled to gait cycle phase during treadmill walking. Cheron et al. (2012) also used the two most representative independent components of the sensorimotor cortex as input for a Dynamic Recurrent Neural Network (DRNN) learning identification toward the two principal components of the 3 elevation angles (foot, shank, and thigh) of one lower limb kinematics which can be easily interpreted by artificial actuators.

EEG signals are usually analyzed by Fourier transforms (FT). Due to the discrete nature of the data analyzed, signals are cut in several windows and processed (Fast Fourier Transform FFT or Short Time Fourier Transform STFT). Although the information extracted by each epoch can provide the evolution through time of the frequency components, other techniques could be more suitable for its time-frequency analysis due to the non-steady nature of EEG signals.

In literature, there are a few examples of these techniques, such as the wavelet transform (Subasi, 2005). However, it needs a good choice of the wavelet mother function which can make this process difficult. In our previous research (Ortiz et al., 2017), we introduced the application of a time-frequency analysis transform, the Hilbert Huang Transform (HHT) (Huang et al., 1998), in an offline scenario for lower limb detection of start and stop of gait cycle based on the ERD/ERS phenomenon. HHT combines a decomposition algorithm Empirical Mode Decomposition (EMD) and a mathematical transform Hilbert Transform (HT). This paper expands our previous research introducing a new transform, the Stockwell Transform (ST) (Stockwell et al., 1996), in order to compare its performance not only with HHT, but also with the FFT. Besides, the study is carried out in an offline and a pseudo-online scenario for better comparison of the techniques. In order to correctly measure the performance of the different proposals, the Weighted Discriminator index (WD) is used (Rodríguez-Ugarte et al., 2017).

The purpose of this work is to show how time-frequency techniques, such as the ST transform, improve the accuracy of the start and the stop detection of gait cycles through the EEG signal analysis, lowering the number of false detections. Sixteen different subjects (eight healthy and eight patients) participated in the research. Data was not only analyzed offline, but pseudo-online as this approach simulates the behavior of the BMI working with an external device in real time.

## 2. Materials and methods

This section provides information about the experimental setup, equipment used for EEG acquisition, motion capture system (MCS) and the data processing.

### 2.1. Experimental setup

Data was collected on sixteen participants. Eight participants (labeled as H1-8) were healthy and did not have any known health issue. They were all right-handed, and were in the age range of 24–29 years (28.2 ± 3.0) at the time of the experiment. Additionally, there were eight patients (labeled as P1-8) of the National Hospital for Spinal Cord Injury in Toledo (Spain), and they were in the age range of 19–71 years (43.7 ± 18.4). This study was carried out in accordance with the recommendations of ethics committee of the Miguel Hernández University of Elche (Spain) with written informed consent from all subjects. All subjects gave written informed consent in accordance with the Declaration of Helsinki. The protocol was approved by the ethics committee of the Miguel Hernández University of Elche (Spain).

Healthy subjects performed ten different trials. However, certain patients due to limitations, tiredness and hardware detection problems completed less trials or some of the trials were not correctly accomplished. Table 1 shows the number of trials considered for analysis by each subject.

**Table 1:** Trials performed by subject.

**Subject**	**H1–H8**	**P1**	**P2**	**P3**	**P4**	**P5**	**P6**	**P7**	**P8**
Total number of trials	10	10	7	6	7	8	9	10	9
Trials used for pseudo-online model	1–6	1–6	1–4	1–4	1–4	1–5	1–6	1–6	1–6
Trials used for pseudo-online testing	7–10	7–10	5–7	5–6	5–7	6–8	7–9	7–10	7–9

**Figure 1:**
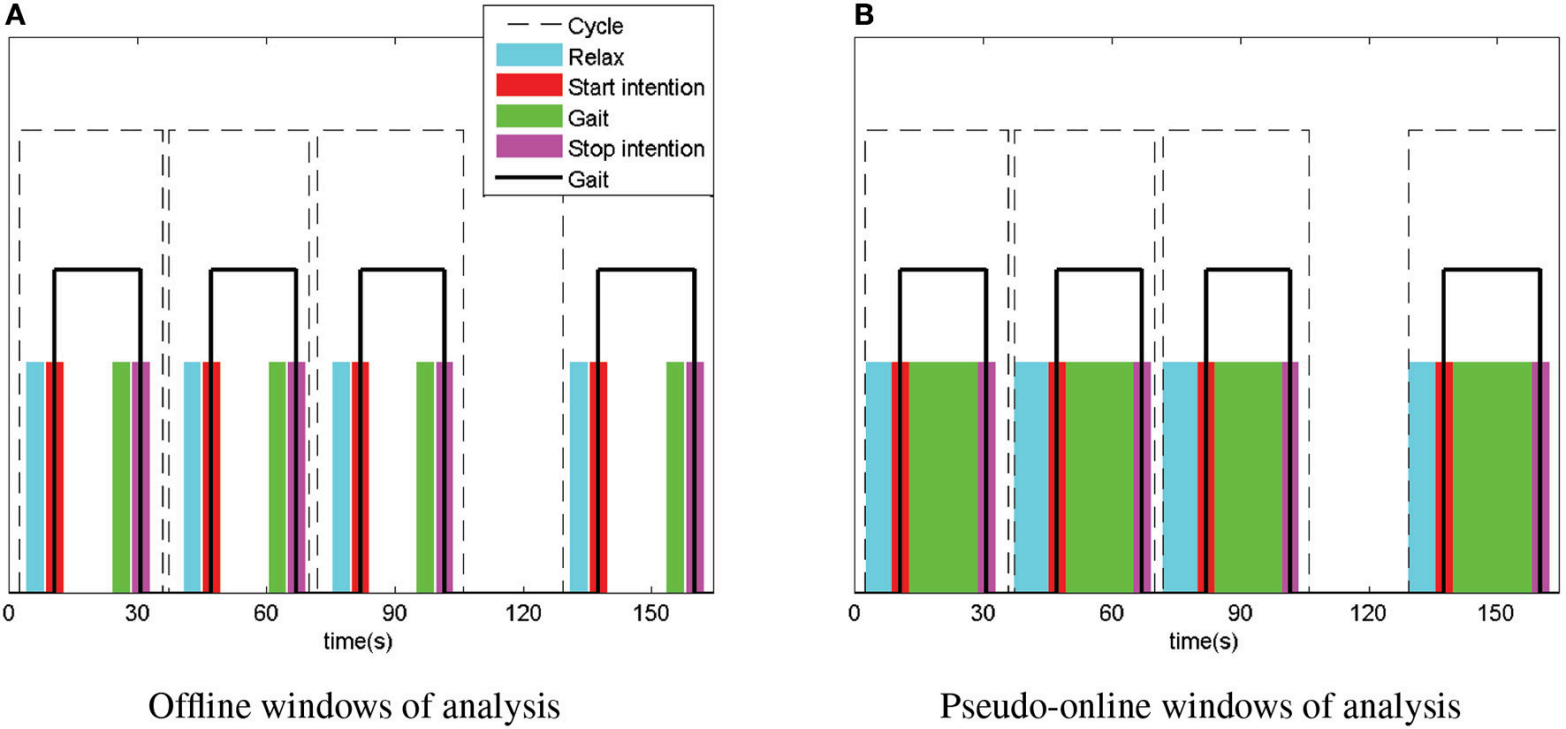
Windows of analysis for offline and pseudo-online models. Time of simulation (broken line) for every trial contains 4 complete gait cycles (solid line). Intention windows (red and magenta) were considered as the active data of the model (state 1). Relax and gait windows (cyan and green) were considered as the non-active data of the model (state 0). The data represented in the figure correspond to the 10th trial of patient P1. **(A)** Offline windows of analysis. **(B)** Pseudo-online windows of analysis.

Each trial consisted of 4 complete gait cycles, each one with two events: start and stop as can be seen in Figure 1. Each cycle followed the next pattern: relax, start intention of gait, gait, stop intention of gait and stop (out of the model analysis, although it can be considered as a relaxed state).

The purpose of the paper is to measure the performance of a BMI that detects the starts or stops of the gait cycle. Hence, two different models were considered, one for the detection of start and other for the detection of stop. The analysis was carried out considering each event (start or stop intention) as the active part of the model (state 1) and the previous state (relax or gait) as the non-active (state 0).

Data processing was not performed in real time. Two different approaches were considered in order to measure the performance of the methods and the classification model: offline and pseudo-online. Pseudo-online analysis simulated the behavior of the tool in real-time conditions. State labels (0 and 1) were defined based on the Inertial Measurement Units (IMU) activation. Active windows had a 4 s duration starting 2 s previously to the IMU activation. Non-active windows were considered before the active windows with a time gap of 0.5 s. In the case of the offline model, active windows had a duration of 4 s, covering the whole previous time of analysis for the pseudo-online model. Differences between the data windows are shown in Figure 1 for both approaches. Nevertheless, data was sampled at 500 Hz and sent every 0.2 s in epochs of 1 s duration to the data processing tools. This allowed to treat the data in a similar way for all the methods tested.

### 2.2. Brain-machine interface

#### 2.2.1. EEG data acquisition

**Figure 2:**
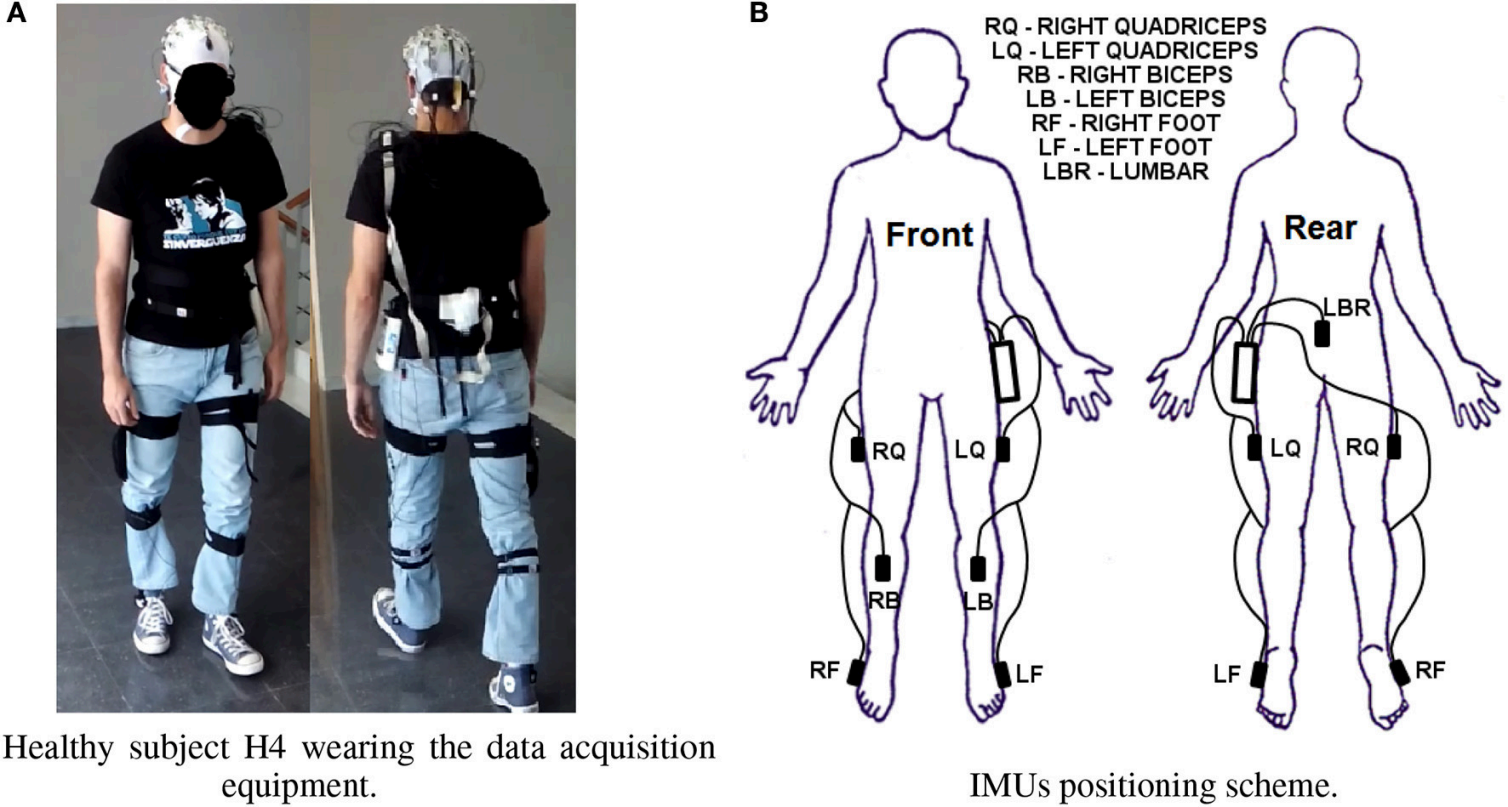
Data acquisition equipment. **(A)** Shows subject H4 wearing the fixing bands for the IMUs, the actiCAP, and Move transmitter. **(B)** Shows a scheme for the positioning of the IMUs from front and rear view. **(A)** Healthy subject H4 wearing the data acquisition equipment. **(B)** IMUs positioning scheme.

**Figure 3:**
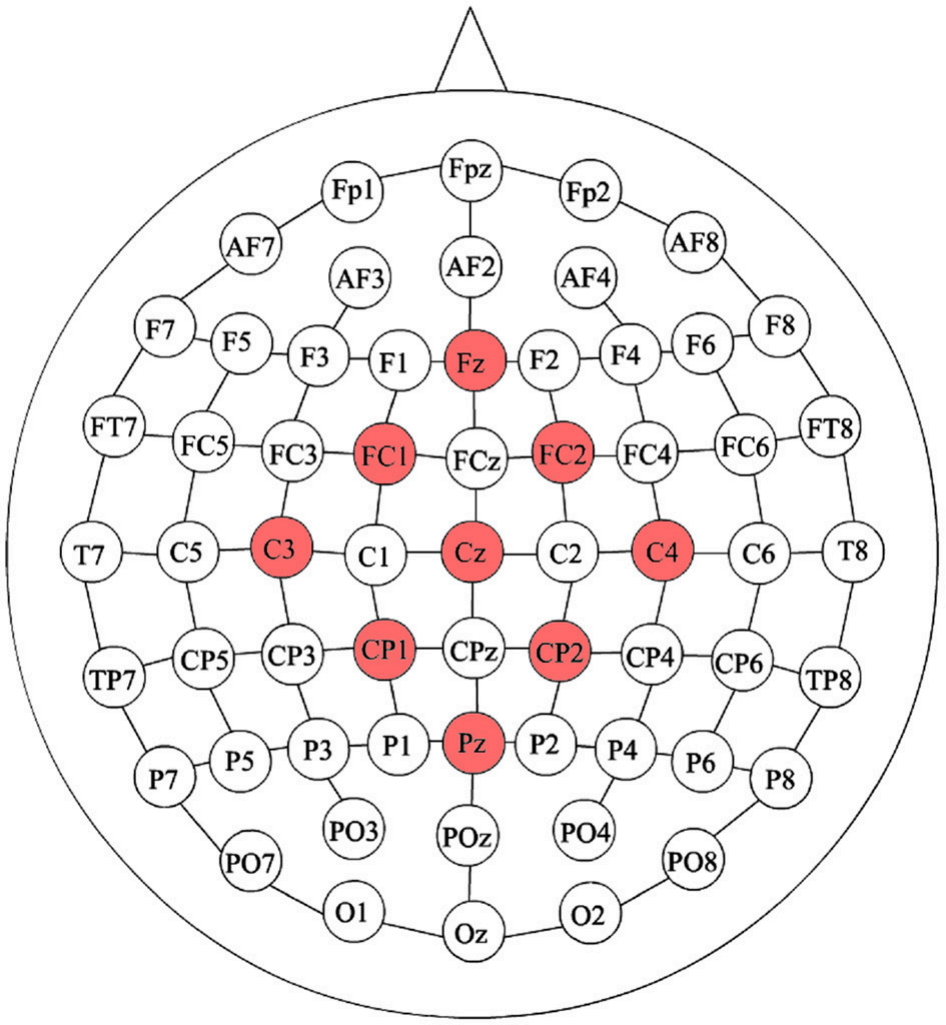
Electrode configuration considered.

EEG signals were recorded using a commercial device developed by Brain Products GmbH (Germany). 31 electrodes were used with the help of an actiCAP for an easier placement. The system registered the EEG signals through the actiCHamp amplifier. They were wireless transmitted by a MOVE transmitter for offline and pseudo-online analyses at a sampling frequency rate of 500 Hz. Electrodes were positioned according to the International 10/10 system. Figure 2A shows healthy subject H4 walking during a trial. Although 31 electrodes were recorded, finally only nine electrodes were used. This is based on previous studies (Hortal et al., 2016b; Ortiz et al., 2017) and preliminary results of the methods tested on this paper for offline scenario. The electrodes chosen were those close to the electrode Cz (Fz, FC1, FC2, C3, Cz, C4, CP1, CP2, and Pz). The reference was positioned on the right ear lobe and the ground on AFz. Figure 3 shows the electrode configuration used.

#### 2.2.2. Motion capture system

For the MCS a Tech MCS manufactured by Technaid S.L. (Spain) was used. The system consisted of seven wireless Inertial Measurement Units (IMUs) located on the following positions: three at each limb (foot, thigh and leg) and one on the lumbar position. Figure 2 shows the exact position of the units. Each IMU had three different types of sensors: an accelerometer, a gyroscope and a magnetometer. Each IMU provided 19 parameters: 9 for rotation, 3 for acceleration, 3 for angular velocity, 3 for magnetic field and 1 for temperature. Rotation parameters were used to detect the initiation and stop of the movement. The data registered by each IMU were acquired through a HUB connected to the PC USB port at a sampling rate of 20 Hz. The MCS mission was to provide the feedback of gait state changes for accuracy calculation of the tests, i.e., the correct detection of the real initiation and stop of the gait.

#### 2.2.3. Preprocessing

Reducing signal to noise ratio is important to improve the feature extraction, so pre-processing of data was needed for each epoch. Several filters were considered.

##### 2.2.3.1. Hardware filter

As this depends on the equipment used for data acquisition, it was the same for all the measurements. The hardware used applied a low pass filter with a cut off frequency of 100 Hz and a notch filter at 50 Hz in order to mitigate the power line interference.

##### 2.2.3.2. Spatial filter

The use of a spatial filter helps to minimize the contribution of the rest of the electrodes to each channel. This way the information of each sensor is better isolated (McFarland et al., 1997). In a previous study (Ortiz et al., 2017) a Laplacian (Lp) and a Common Average Reference (CAR) spatial filter were compared during offline analysis, obtaining Lp filter a better outcome in average than CAR for both event detection (7.8 % better comparison index for the FFT and 6.3 % better for the EMD). Therefore, Lp was the spatial filter used in this study. It aims to subtract the contribution of the rest of electrodes based on distance. Equation 1 shows how the filtered voltage is calculated for electrode *i*.

(1) $$\begin{array}{r} V_{i}^{Lp}=V_{i}-\sum_{i\neq j}g_{ij}\cdot V_{j} \end{array}$$

Where $V_{i}^{Lp}$ represents the voltage after filtering, *V*_*j*_ is the voltage without filtering, *j* = 1:31 and *g*_*ij*_:

(2) $$\begin{array}{r} g_{ij}=\frac{\frac{1}{d_{ij}}}{\underset{i\neq j}{\sum }\frac{1}{d_{ij}}} \end{array}$$

With *d*_*ij*_ representing the distance between the electrodes *i* and *j* based on the three dimensional Euclidean method.

##### 2.2.3.3. Frequency filter

The application of the frequency filter depends on the algorithm or mathematical transform used for data analysis. In this research it was only used before the FFT processing. Based on our previous study (Ortiz et al., 2017) a 4th order Butterworth high-pass filter with a cut off frequency of 0.2 Hz was used to extract the DC component of the signal. HHT and ST methods are not affected by the low pass filter, so it would only increase the computing time without an improvement of the results.

#### 2.2.4. Processing

Three different processing tools were tested to obtain the feature vector of the signal: FFT, HHT, and ST. The three methods were used to extract the data characteristics from the same frequency bands: 8–13, 13–32, and 32–50 Hz. They are related to alpha, beta (ERD/ERS phenomenon) and gamma (attentive focus) bands. Therefore, for all processing methods, each electrode provides three features. Following paragraphs describe the nature of these methods.

##### 2.2.4.1. Fast fourier transform

Fourier transform (Bracewell and Bracewell, 1986) is one of the most extended tools in signal processing. The non-steady state nature of EEG signals is minimized thanks to the analysis of the signal in epochs. FFT provides information of the evolution in time (different epochs moving every 0.2 s) of the harmonic content. For each epoch and electrode, the harmonic content in each band was computed.

##### 2.2.4.2. Hilbert huang transform

HHT was developed by Huang et al. (1998) as an improvement to Hilbert Transform (HT) application. It consists of a sifting process based on the envelopes of data. This process, called Empirical Mode Decomposition (EMD) separates a signal in several Intrinsic Mode Functions (IMFs). The process can be described as follows:

1. Find the local extrema of *x*(*t*).
2. Find the maximum envelope *e*_+_(*t*) of *x*(*t*) by fitting a natural cubic spline through the local maxima. Then, repeat this step to find the minimum envelope, *e*_−_(*t*), by using the local minima.
3. Compute an approximation to the local average: *m*(*t*) = (*e*_+_(*t*) + *e*_−_(*t*))/2.
4. Find the proto-mode function: *p*_*i*_(*t*) = *x*(*t*) − *m*(*t*).
5. Check if *p*_*i*_(*t*) is an IMF:
  - The number of extrema and the number of zero crossings may differ by no more than one.
  - The local average is zero. The thresholds chosen to set this condition are critical to avoid over or undertraining. In this research the stopping criteria thresholds of Rilling et al. (2003) were followed.
  - To avoid the extraction of accidental IMFs, the conditions must be accomplished in at least two to three consecutive iterations (three in our case).

6. If *p*_*i*_(*t*) is not an IMF, repeat the EMD sifting process by setting: *x*(*t*) = *p*_*i*_(*t*). If *p*_*i*_(*t*) is an IMF then set: *IMF*_*i*_(*t*) = *p*_*i*_(*t*).

Every *IMF*_*i*_(*t*) is supposed to be monotonic if EMD is successfully applied. Therefore, *IMF*_*i*_(*t*) and its HT are orthogonal and instantaneous amplitude *a*(*t*) and pulsation ω_*i*_(*t*) can be computed through the analytical complex function *z*_*i*_(*t*) analysis (Huang et al., 1998) of every mode:

(3) $$\begin{array}{r} z_{i}\left(t\right)=IMF_{i}\left(t\right)+jHT\left(IMF_{i}\left(t\right)\right)=a_{i}\left(t\right)e^{j\phi_{i}\left(t\right)} \end{array}$$

(4) $$\begin{array}{r} \omega_{i}\left(t\right)=\frac{d\phi_{i}\left(t\right)}{dt} \end{array}$$

**Figure 4:**
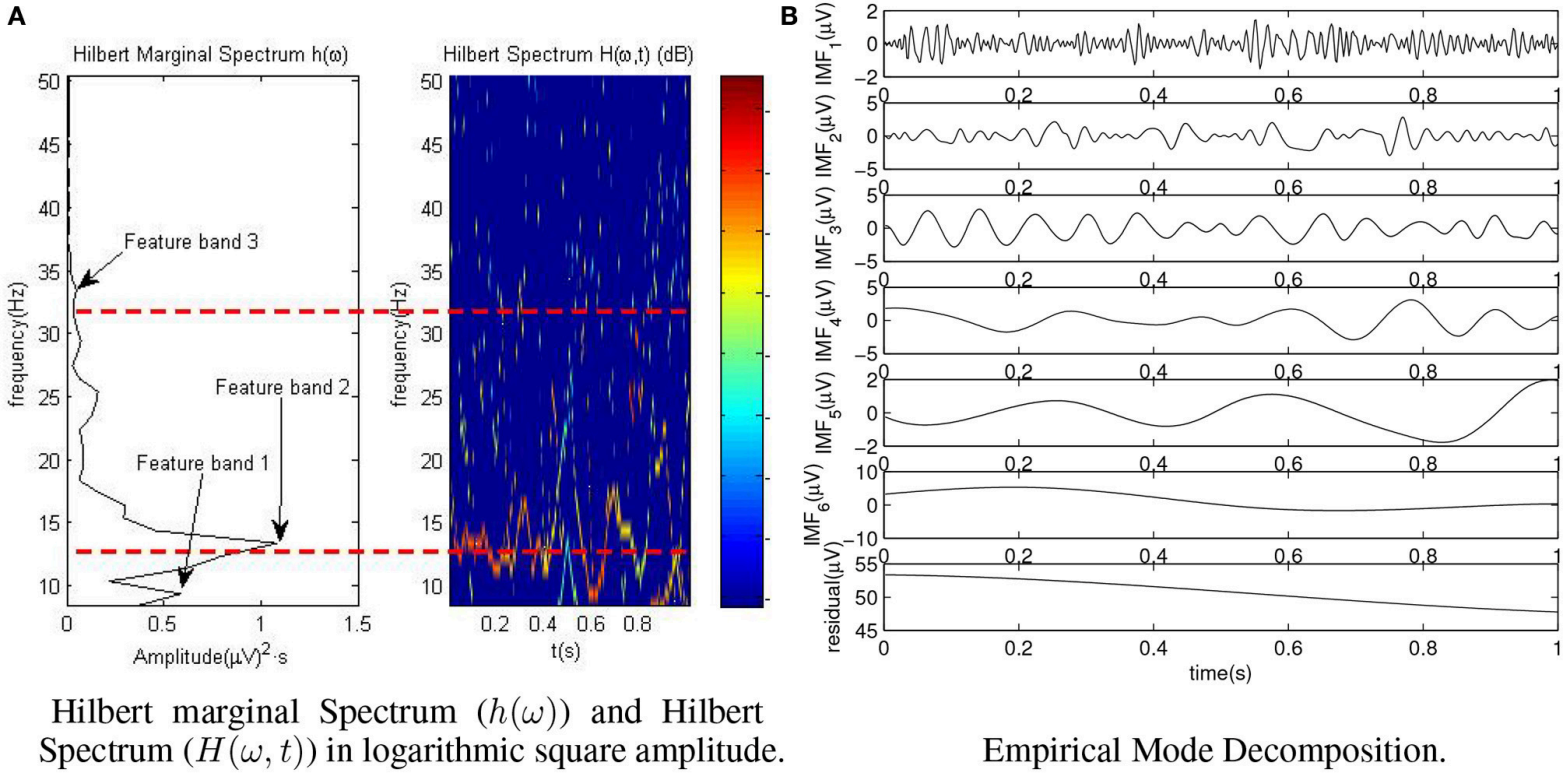
Representation of Hilbert Spectrum (*H*(ω, *t*)) and IMFs of an epoch for the healthy subject H4. Hilbert Spectrum is represented in logarithmic scale for better visualization. Marginal Spectrum *h*(ω) is shown as square amplitude per time. **(A)** Hilbert marginal Spectrum (*h*(ω)) and Hilbert Spectrum (*H*(ω, *t*)) in logarithmic square amplitude. **(B)** Empirical mode decomposition.

Once all the modes are extracted (see Figure 4B for an example), Hilbert Spectrum *H*(ω, *t*) (Huang et al., 1998) is calculated based on *a*_*i*_(*t*) and ω_*i*_(*t*) for all the modes. *H*(ω, *t*) is computed as a function of energy (square amplitude) by frequency and time (right part of Figure 4A). As data volume of *H*(ω, *t*) can be too large, the Hilbert Marginal Spectrum *h*(ω) is computed as Equation (5). This is carried out for each epoch as:

(5) $$\begin{array}{r} h\left(\omega \right)=\overset{t_{2}}{\underset{t_{1}}{\int }}H\left(\omega ,t\right)dt \end{array}$$

Being *t*_2_ − *t*_1_ = 1*s*.

For each electrode and epoch the peak value of each frequency band is extracted as one feature of data. See Figure 4A for a clearer representation of *h*(ω) features. Notice that in Figure 4A *H*(ω, *t*) is represented in logarithmic square amplitude, instead of square amplitude, for a better visualization.

However, there are difficulties related to the algorithm nature of the EMD. The sifting process is sensitive to the thresholds defined in the algorithm (Rilling et al., 2003) and the sampling frequency (Rilling and Flandrin, 2006). Besides, it can be hard to extract components that present similar tones (Rilling et al., 2003; Rilling and Flandrin, 2008) which can affect to the quality of the *H*(ω, *t*) due to the lack of orthogonality of some of the modes.

##### 2.2.4.3. Stockwell transform

Stockwell transform, also known as S-Transform (ST), was developed as a time-frequency decomposition tool (Stockwell et al., 1996). It overcomes some of the disadvantages of Short Time Fourier Transform (STFT) (better time-frequency resolution) based on a scalable localizing Gaussian window. It is defined as:

(6) $$\begin{array}{r} S\left(\tau ,f\right)=\int_{-\infty }^{+\infty }x\left(t\right)\frac{\left|f\right|}{\sqrt{2\pi }}e^{-\frac{{\left(\tau -t\right)}^{2}f^{2}}{2}}e^{-j2\pi ft} \end{array}$$

One of the properties of ST is to define multiple frequency voices as one dimensional functions of time scale (τ) and frequency (*f*_*i*_):

(7) $$\begin{array}{r} S\left(\tau ,f_{i}\right)=A\left(\tau ,f_{i}\right)e^{j\phi \left(\tau ,f_{i}\right)} \end{array}$$

Due to the orthogonal nature of voice functions, local frequency and amplitude can be computed which allows to obtain *H*(ω, *t*). Once it is created for each epoch, *h*(ω, *t*) is calculated in the same way that was explained for HHT in paragraph 2.2.4.2 and Figure 4A, obtaining the three features per electrode based on the *h*(ω) peaks per band. ST and HHT are similar in the way the features are extracted, but different in the way *H*(ω, *t*) is computed. The main advantage of ST is its analytical nature which makes it not dependable of any thresholds. However, although it improves the frequency resolution of FFT, it has still a worse frequency resolution the higher the frequency is. As the frequency bands related to the characteristics (8–50 Hz) are far from the Nyquist frequency (250 Hz), this is not a problem for our study.

#### 2.2.5. Post-processing

Once the features were extracted per each electrode (9 × 3 = 27 data vector per 1 s epoch). Two different tests were done: offline and pseudo-online.

First, it was necessary to create a model for the later test data identification. Each participant had this way four different models associated: one for type of event detection (start or stop) and one for approach (offline or pseudo-online). The model allowed to identify testing epochs as non-active (0 label for rest or gait) and active intention (1 label for start intention or stop intention).

##### 2.2.5.1. Classifier

The classifier chosen was the Support Vector Machine (SVM) algorithm. The SVM is based on hyperplane separation by maximizing the margin between the nearest points of different classes (Steinwart and Christmann, 2008). SVM combined with non-linear kernels, such as the radial basis used for this research, results in a robust method (Hortal et al., 2016a; Sburlea et al., 2017). Other alternative classifiers such as Self-organizing maps (SOM) and Linear Discrimination Analysis (LDA) were also considered and tested for some of the healthy subjects at the first steps of the research. However, the higher time of processing required for the model creation in the case of the SOM classifier and the overall better results obtained by SVM were the reason to select it. In order to limit the volume of data presented, only SVM results are shown on this paper. The model creation and evaluation was carried out in a different way for offline and pseudo-online approaches.

In the case of offline analysis, subjects were evaluated by leave-one-out cross-validation. This means that for each participant, one trial was used for validation and the rest of the trials for modeling. For instance, in the case of a subject with ten trials registered, ten different models of nine trials were performed for start intention and another ten for stopping. The ten test trials were evaluated for each one of their models and finally the results were averaged.

For pseudo-online tests, the first trials were used to create the model and the last ones to test it as if they were processed in real time. Therefore, evaluation was carried out without leave-one-out cross-validation. In the case of healthy participants, the ratio was six tests for modeling and four for testing (6/4 ratio). As the number of trials for patients was inferior to ten in some cases, the ratio presented minor differences, e.g., P2 had a 4/3 ratio. Table 1 shows the trials used for the model creation and testing by user. The indices associated with the test trials of each subject were also averaged.

**Figure 5:**
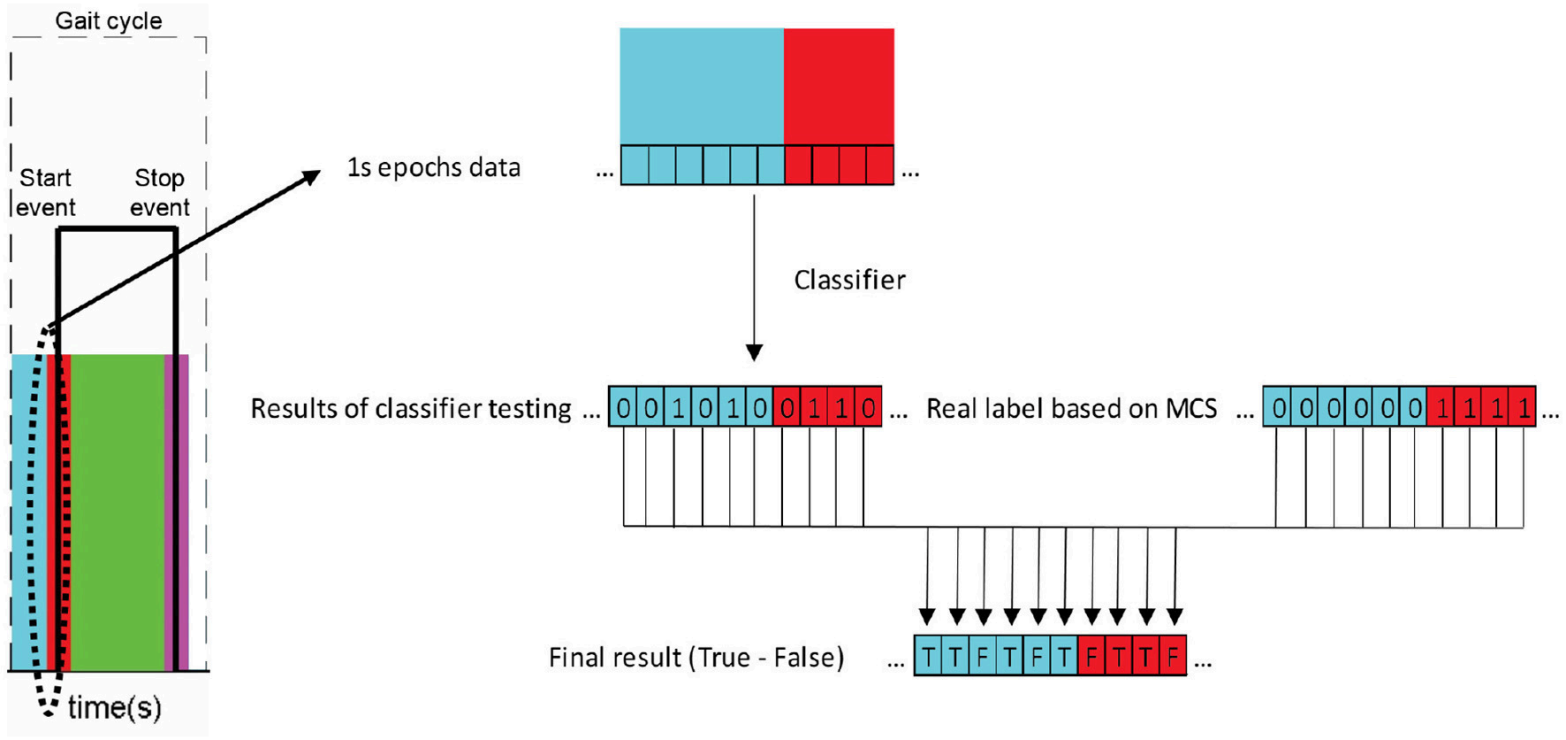
Scheme of evaluation of data epochs tested on the classifier.

For evaluation, each epoch, formed by a 27 features vector, was tested over the classifier and a label 0 or 1 was returned. This label was compared with the true nature of the epoch, based on the MCS data, and a result of a true (T) or false detection (F) was registered. The process is shown in Figure 5. This true and false vector was used afterwards for the index evaluation.

##### 2.2.5.2. Evaluation indices

The most common way to evaluate the results is using the following indices: True Positive Rate (TPR), False Positive per Minute (FP/min) and Accuracy (Acc).

TPR indicates the percentage of true start or stop intention events detected. This was evaluated only for the active windows (red or magenta in Figure 1). The evaluation of a true event detection was a bit different for the two analysis. Offline, a number of *T* > *F* was enough to consider a true event, while on pseudo-online, more than five consecutive *T* were needed to consider the whole event as true. A single trial TPR would be defined as:

(8) $$\begin{array}{r} TPR=\frac{Number\;of\;true\;event\;detections}{Number\;of\;true\;events} \end{array}$$

Accuracy appoints how many start or stop intentions detected were really a true detection. This means that it has to be evaluated for active and non-active windows. In the case of non-active windows, a false detection was achieved when *F* > *T* (offline) or more than five consecutive *F* were accounted (pseudo-online). In active windows, the calculation of a detection was the same as TPR. The Acc of a trial would be:

(9) $$\begin{array}{r} Acc=\frac{Number\;of\;true\;event\;detections}{Number\;of\;total\;event\;detections} \end{array}$$

FP/min indicates the number of false detections per minute. It is an important index, because a high number would result in a disturbing operation of the mobility assistant device, in which the mechanism would be activated without the real subject desire. FP/min were computed for non-active windows when *F* > *T* in the case of offline analysis. However, pseudo-online analysis is a bit different. As it tries to simulate the real-time behavior of the tool and several FP can occur during non-active windows, it was necessary to compute all the false activations and not only one by event. This way, a FP was computed each time more than five consecutives *F* were detected. The rest or gait windows (non-active) for the offline scenario have the same length than active windows: 4 s, i.e., 4/60 min, while for the pseudo-online scenario expand for the whole rest or gait time previous to the 0.5 gap between non-active and active windows per event. This can be seen in Figure 1B as cyan or green windows for the start and the stop models. FP/min can be expressed as:

(10) $$\begin{array}{r} FP/min=\frac{Number\;of\;false\;activations}{Rest\;or\;gait\;time\;windows\;in\;minutes} \end{array}$$

It is important to remark that the three indices are necessary in order to correctly analyze the results. For instance, a trial test with 100% Acc and 0 FP/min could seem a perfect one, but it may only indicate that one of the four events of a trial was detected, being in this case the TPR only 25%. Although the previous indices provide a good information of the results, it can be difficult to compare them based on three independent indices. Consequently, in a previous research, a unified index called Weighted Discriminator (WD) was developed (Rodríguez-Ugarte et al., 2017). WD takes into account TPR, Acc and the False Positive Ratio (FPR) which provides the ratio of false positives per event:

(11) $$\begin{array}{r} WD=0.4\cdot TPR+0.6\cdot Acc-FPR \end{array}$$

with TPR and FPR in p.u. and:

(12) $$\begin{array}{r} FPR=FP/min\cdot Duration\;of\;a\;single\;FP\;in\;mins \end{array}$$

Being the duration for the offline analysis 4/60 min and 1/60 min for the pseudo-online one (equivalent to five consecutive detections represented by the gap of 0.2 s). WD can oscillate from a perfect performance value of 1 to the worst if value is −1. Therefore, WD acts as a comprehensive index to compare the performance of the different tests.

## 3. Results

Results were obtained for the sixteen subjects and the offline and pseudo-online analysis. The comprehensive WD index was calculated from the determination of TPR, FP/min and Acc indices in order to evaluate the performance of the BMI in every case. WD index was statistically analyzed. A Shapiro-Wilks (S-W) test of normality was applied in order to detect outliers (Ghasemi and Zahediasl, 2012) and a factorial multivariate analysis of variance (MANOVA) was carried out to detect the significant differences between methods (ST/HHT/FFT), type of subject (healthy/patient) and type of event (start/stop) with the help of SPSS (Field, 2009).

### 3.1. Offline analysis

As previously stated, offline analysis was carried out by leave-one-out cross-validation technique. WD acts as a measurement of the BMI performance. It was computed for each method, subject and model. This can be seen in Tables 2–4.

**Table 2:** Offline results for the sixteen subjects.

**Subject**	**Method**	**Start**				**Stop**			
		**TPR (%)**	**FP/min**	**Acc (%)**	**WD**	**TPR (%)**	**FP/min**	**Acc (%)**	**WD**
H1	ST	87.50	3.75	77.83	**0.50**	75.00	1.13	92.17	**0.76**
	HHT	77.50	4.50	74.79	0.38	82.50	1.88	89.00	0.71
	FFT	65.00	3.75	71.50	0.38	80.00	2.63	85.17	0.61
H2	ST	87.50	2.63	88.00	**0.66**	85.00	1.50	91.50	**0.76**
	HHT	77.50	4.13	70.67	0.39	75.00	1.88	87.67	0.67
	FFT	70.00	3.00	79.33	0.51	80.00	2.25	84.83	0.64
H3	ST	82.50	2.63	84.83	**0.62**	82.50	0.75	95.50	**0.84**
	HHT	65.00	6.75	60.17	0.06	75.00	1.88	89.67	0.68
	FFT	70.00	5.25	75.21	0.29	77.50	2.63	82.00	0.58
H4	ST	95.00	1.50	93.33	**0.81**	87.50	0.38	98.00	**0.91**
	HHT	82.50	2.63	86.71	0.63	72.50	0.38	98.00	0.85
	FFT	80.00	1.13	93.00	0.78	85.00	2.25	88.17	0.68
H5	ST	88.00	2.10	87.14	**0.70**	82.00	1.50	92.14	**0.76**
	HHT	78.00	3.60	76.95	0.47	74.00	1.80	85.17	0.66
	FFT	72.00	2.10	85.14	0.62	84.00	3.60	83.06	0.53
H6	ST	90.00	2.70	84.79	0.64	66.00	0.90	93.33	**0.75**
	HHT	72.00	3.00	84.10	0.54	44.00	1.20	90.00	0.62
	FFT	82.00	1.80	89.31	**0.71**	58.00	2.70	78.50	0.48
H7	ST	86.00	3.90	81.25	**0.51**	82.00	0.60	96.00	**0.85**
	HHT	58.00	7.50	54.76	-0.06	50.00	3.60	67.48	0.30
	FFT	66.00	3.90	72.50	0.37	66.00	3.30	77.14	0.45
H8	ST	94.00	3.00	84.23	0.63	82.00	0.60	96.33	**0.86**
	HHT	64.00	4.20	68.52	0.32	38.00	0.30	86.67	0.65
	FFT	82.00	1.20	93.00	**0.79**	72.00	1.80	82.08	0.63
P1	ST	85.00	0.38	97.50	**0.89**	60.00	3.38	85.83	**0.47**
	HHT	77.50	1.50	84.00	0.69	47.50	1.88	70.50	0.46
	FFT	85.00	1.50	92.00	0.77	60.00	3.00	77.00	0.45
P2	ST	53.57	2.68	70.48	**0.41**	89.29	4.29	83.16	**0.50**
	HHT	96.43	9.64	64.49	-0.03	46.43^*^	7.50^*^	26.19^*^	-0.28^*^
	FFT	67.86	4.82	65.24	0.26	71.43	5.36	73.81	0.28
P3	ST	66.67	5.63	66.67	0.20	50.00	1.88	73.61	**0.49**
	HHT	54.17	6.25	47.50	-0.02	50.00	3.75	77.78	0.35
	FFT	58.33	4.38	69.44	**0.29**	50.00	5.63	56.94	0.07
P4	ST	92.86	2.68	86.90	**0.67**	57.14	2.68	68.57	0.42
	HHT	78.57	2.68	81.67	0.58	50.00	1.07	93.57	**0.67**
	FFT	78.57	2.68	84.52	0.60	57.14	3.75	71.19	0.34
P5	ST	90.63	2.34	89.64	**0.71**	71.88	1.41	90.63	**0.71**
	HHT	84.38	4.69	75.21	0.40	68.75	1.88	90.83	0.66
	FFT	84.38	3.75	80.68	0.51	68.75	2.34	85.42	0.59
P6	ST	84.00	3.00	83.93	0.59	72.00	2.40	87.52	**0.61**
	HHT	76.00	6.60	63.88	0.14	56.00	2.40	70.64	0.45
	FFT	86.00	3.00	84.57	**0.60**	68.00	2.10	83.81	0.60
P7	ST	72.00	5.10	69.72	0.28	64.00	3.00	79.00	**0.48**
	HHT	46.00	4.50	61.79	0.18	46.00	3.00	59.31	0.29
	FFT	68.00	4.50	70.42	**0.32**	58.00	2.70	77.64	0.47
P8	ST	80.00	2.00	89.42	0.69	91.11	1.67	94.44	0.79
	HHT	66.67	1.00	94.44	**0.75**	95.56	0.33	98.15	**0.94**
	FFT	86.67	3.00	82.01	0.59	93.33	1.00	94.44	0.86

The data represent the average value of the trials (up to ten by subject) obtained by leave-one-out cross-validation technique for the different evaluation indices. Outliers identified by SPSS are marked by

* *. Best WD results by subject are marked in bold for the start and stop models*.

The S-W test indicated that P2 was an outlier for the HHT stop model (*p* < 0.05) and, as a consequence, it was not considered for the stop model.

In order to carry out a MANOVA test, it is needed to assess if the variances between groups are equal (assumption of sphericity). This test is known as Mauchly's test. In this case, assumption of sphericity was not violated (*p* > 0.05).

Then, it was performed the MANOVA analysis. The interaction between methods and type of event in the test of within-subjects presented significant differences [*F*_(2, 54)_ = 10.80, *p* < 0.001, η^2^ = 0.29]. In order to see the cause of this, a pairwise comparison using Bonferroni confidence interval adjustment was performed. ST and FFT had no significant differences (*p* > 0.05), but HHT did (*p* < 0.01) for the start and stop models. On the other hand, the interaction, in the test of within-subjects, between methods and type of subject presented no significant differences (*p* > 0.05). The interaction between type of event and type of subjects in the test of between-subjects was also not significant (*p* > 0.05).

Regarding the WD value, ST obtained the best results for both start and stop models (bold text in Table 3). Although, there were no significant differences depending on the type of subject, WD results for healthy users were higher in average (bold text for average in Table 4). ST was also the method with the highest WD for both type of subjects as Table 4 shows, but with a lower difference with the other methods for patients than for healthy subjects. HHT performance was irregular with the lowest WD results for the start model and the highest standard deviation in Tables 3, 4.

**Table 3:** Offline results by method.

**Method**	**Start**				**Stop**			
	**TPR (%)**	**FP/min**	**Acc (%)**	**WD**	**TPR (%)**	**FP/min**	**Acc (%)**	**WD**
ST	83.45 ± 10.98	2.88 ± 1.27	83.48 ± 8.54	**0.60** ± **0.18**	73.88 ± 12.22	1.59 ± 0.93	88.97 ± 8.74	**0.70** ± **0.16**
HHT	72.14 ± 12.59	4.57 ± 2.30	71.85 ± 12.71	0.34 ± 0.27	61.65 ± 16.94	1.82 ± 1.08	83.63 ± 11.74	0.60 ± 0.19
FFT	75.11 ± 8.94	3.11 ± 1.31	80.49 ± 8.98	0.52 ± 0.19	70.51 ± 12.53	2.78 ± 1.05	80.49 ± 8.53	0.53 ± 0.18
Average	76.90 ± 11.75	3.52 ± 1.82	78.61 ± 11.20	0.49 ± 0.24	68.68 ± 14.69	2.06 ± 1.12	84.36 ± 10.19	**0.61** ± **0.19**

*The data represent the average value ±, standard deviation by method for the subjects. P2 was not considered for the stop model as it was detected as outlier. Best results for start and stop models appear in bold*.

**Table 4:** WD Offline results by method and type of subject.

**Method**	**Healthy**		**Patient**	
	**Start**	**Stop**	**Start**	**Stop**
ST	**0.64** ± **0.10**	**0.81** ± **0.13**	**0.56** ± **0.24**	**0.57** ± **0.14**
HHT	0.34 ± 0.23	0.64 ± 0.16	0.33 ± 0.31	0.55 ± 0.23
FFT	0.56 ± 0.20	0.58 ± 0.09	0.49 ± 0.18	0.48 ± 0.24
Average	**0.51** ± **0.22**	**0.68** ± **0.14**	0.46 ± 0.26	0.53 ± 0.20

*The data represent the average WD value ± standard deviation by method and type of subject: healthy or patient. P2 was not considered for the stop model as it was detected as outlier. Best results in bold*.

### 3.2. Pseudo-online analysis

The actual application of the BMI is in a real-time situation where the patient is trying to activate the motion device with the BMI output. As the trials were acquired before the ST and HHT implementation by the authors, a pseudo-online approach was adopted to overcome this issue. In a pseudo-online scenario, it is simulated that epochs are processed as they are acquired. First trials were used for modeling, as stated in subsection 2.2.5.1, and the rest of them were reserved for testing (Table 1). It is important to notice that, FP/min was calculated in a different way as several false activations can be registered in real-time non-active windows. Therefore, this approach bring us a more realistic outcome of the BMI behavior, while offline tests gives information of the global performance when applying the different methods. A bad trial performance of a subject had a more relevant influence over the results, because not only an inferior number of trials is considered, but false detections can be multiple for each event.

Table 5 provides the TPR, FP/min, Acc, and WD results for the different methods and subjects whereas Tables 6, 7 show the average WD value for the three methods and type of subject. The results vary from the offline tests as there were differences in the way a detection was computed and the number of trials used for testing. This variation is more noticeable in the case of the number of FP/min, as a comparison between offline and pseudo-online tables shows. However, as the WD takes into account the FPR and the FP time length varies, WD still acts as a good index for the method comparison.

**Table 5:** Pseudo-online results for the sixteen subjects.

**Subject**	**Method**	**Start**				**Stop**			
		**TPR (%)**	**FP/min**	**Acc (%)**	**WD**	**TPR (%)**	**FP/min**	**Acc (%)**	**WD**
H1	ST	81.25	7.68	66.67	**0.56**	93.75	3.99	79.52	**0.79**
	HHT	68.75	6.43	58.75	0.52	68.75	3.36	75.18	0.67
	FFT	68.75	7.27	66.19	0.55	81.25	3.91	78.04	0.73
H2	ST	100.00	5.14	83.57	**0.82**	100.00	8.86	64.09	0.64
	HHT	56.25	4.10	55.36	0.49	68.75	0.65	93.75	**0.83**
	FFT	75.00	3.97	73.87	0.68	100.00	12.05	58.69	0.55
H3	ST	93.75	13.16	66.98	**0.56**	81.25	1.35	92.26	**0.86**
	HHT	68.75	10.27	59.38	0.46	43.75	0.00	75.00	0.63
	FFT	93.75	14.76	57.78	0.48	62.50	0.69	91.67	0.79
H4	ST	100.00	0.00	100.00	**1.00**	81.25	0.00	100.00	**0.93**
	HHT	62.50	7.44	40.21	0.37	25.00	0.00	75.00	0.55
	FFT	75.00	1.80	82.50	0.77	56.25	1.89	68.75	0.61
H5	ST	85.00	9.06	75.70	0.64	85.00	1.83	92.26	**0.86**
	HHT	75.00	4.34	86.31	**0.75**	55.00	3.64	66.67	0.56
	FFT	75.00	8.80	80.31	0.64	70.00	4.32	56.52	0.55
H6	ST	70.00	1.78	78.33	**0.72**	95.00	3.05	81.26	**0.82**
	HHT	65.00	4.66	73.61	0.62	35.00	3.03	52.50	0.40
	FFT	70.00	3.56	78.41	0.69	80.00	8.33	53.84	0.50
H7	ST	70.00	1.93	82.14	**0.74**	85.00	2.69	83.33	**0.80**
	HHT	30.00	7.74	27.68	0.16	40.00	3.60	56.25	0.44
	FFT	50.00	4.29	49.43	0.43	55.00	2.69	70.42	0.60
H8	ST	90.00	4.06	67.38	**0.70**	100.00	1.75	83.33	**0.87**
	HHT	50.00	3.45	50.00	0.44	55.00	2.73	57.50	0.52
	FFT	85.00	5.43	61.81	0.62	95.00	2.42	78.82	0.81
P1	ST	100.00	3.58	84.18	0.85	31.25	0.00	100.00	**0.73**
	HHT	100.00	2.13	89.58	**0.90**	37.50	0.00	75.00	0.60
	FFT	93.75	3.30	87.61	0.85	25.00	0.00	50.00	0.40
P2	ST	50.00	2.62	72.22	**0.59**	91.67	3.54	75.93	**0.76**
	HHT	100.00	21.42	60.58	0.41	100.00	40.14	36.25	-0.05
	FFT	33.33	3.33	35.71	0.29	58.33	2.42	77.78	0.66
P3	ST	62.50	0.00	100.00	0.85	87.50	13.33	30.30	0.31
	HHT	75.00	0.00	100.00	**0.90**	62.50	11.99	25.71	0.20
	FFT	50.00	0.00	100.00	0.80	75.00	13.28	38.87	**0.31**
P4	ST	83.33	0.00	100.00	**0.93**	91.67	18.04	37.87	0.29
	HHT	58.33	3.61	75.00	0.62	66.67	4.65	59.44	**0.55**
	FFT	41.67	0.00	100.00	0.77	66.67	8.51	42.73	0.38
P5	ST	83.33	4.72	75.56	**0.71**	50.00	1.65	74.29	0.62
	HHT	75.00	5.62	65.48	0.60	25.00	4.03	33.33	0.23
	FFT	50.00	1.23	91.67	0.73	58.33	1.46	69.05	**0.62**
P6	ST	73.33	2.85	79.17	**0.72**	86.67	3.04	79.74	**0.77**
	HHT	66.67	5.66	65.61	0.57	80.00	16.13	35.20	0.26
	FFT	40.00	5.00	57.62	0.42	73.33	11.20	38.33	0.34
P7	ST	65.00	6.82	61.16	**0.51**	85.00	11.78	50.87	**0.45**
	HHT	35.00	2.08	48.33	0.40	60.00	11.43	32.91	0.25
	FFT	35.00	3.95	42.50	0.33	45.00	3.23	59.72	0.48
P8	ST	80.00	7.41	73.61	0.64	100.00	0.85	96.67	**0.97**
	HHT	53.33	0.00	100.00	**0.81**	86.67	0.85	95.24	0.90
	FFT	86.67	6.22	76.67	0.70	73.33	2.54	89.26	0.79

*Data represent the average value of the tested trials. No outliers were identified by SPSS. Best WD results appear in bold*.

**Table 6:** Pseudo-online results by method.

**Method**	**Start**				**Stop**			
	**TPR (%)**	**FP/min**	**Acc (%)**	**WD**	**TPR (%)**	**FP/min**	**Acc (%)**	**WD**
ST	80.47 ± 14.63	4.43 ± 3.67	79.17 ± 12.19	**0.72** ± **0.14**	84.06 ± 18.40	4.73 ± 5.34	76.36 ± 20.95	**0.72** ± **0.21**
HHT	64.97 ± 18.96	5.56 ± 5.04	65.99 ± 20.59	0.56 ± 0.20	56.85 ± 21.68	6.64 ± 10.11	59.06 ± 21.90	0.47 ± 0.25
FFT	63.93 ± 20.96	4.56 ± 3.64	71.38 ± 19.46	0.61 ± 0.17	67.19 ± 18.40	4.93 ± 4.28	63.91 ± 16.82	0.57 ± 0.16
Average	69.79 ± 19.54	4.85 ± 4.11	72.18 ± 18.26	**0.63** ± **0.18**	69.37 ± 22.24	5.44 ± 6.95	66.44 ± 20.92	0.59 ± 0.23

*The data represent the average value ± standard deviation by method for the sixteen subjects. Best results for WD start and stop models appear in bold*.

**Table 7:** WD pseudo-online results by method and type of subject.

**Method**	**Healthy**		**Patient**	
	**Start**	**Stop**	**Start**	**Stop**
ST	**0.72** ± **0.14**	**0.82** ± **0.09**	**0.73** ± **0.14**	**0.61** ± **0.24**
HHT	0.48 ± 0.17	0.58 ± 0.14	0.65 ± 0.20	0.37 ± 0.30
FFT	0.61 ± 0.11	0.64 ± 0.12	0.61 ± 0.23	0.50 ± 0.17
Average	0.60 ± 0.17	**0.68** ± **0.15**	**0.66** ± **0.19**	0.49 ± 0.25

*The data represent the average WD value ± standard deviation by method and type of subject: healthy or patient. Best results in bold*.

The S-W test of normality was passed for all the models (*p* > 0.05) and no outliers were detected. Hence, the sixteen subjects were considered for pseudo-online analysis.

Mauchly's test indicated that the assumption of sphericity was violated (*p* < 0.05). Therefore, it was needed to apply the corrector factor with the highest power, in this case Huynh-Feldt.

For the MANOVA analysis, the test of within-subjects effects presented no significant differences (*p* > 0.05) for the interaction between methods and type of event, and for the interaction between the methods and the type of subject, applying for both cases the corresponding corrector factor of Huynh-Feldt. The interaction between type of event and type of subject in the test of between-subjects effects presented significant differences [$F_{\left(1,\;28\right)}=6.34,p<0.05,\eta^{2}=0.185$]. The pairwise comparison using Bonferroni confidence interval adjustment did not detect differences for healthy subjects (*p* > 0.05), but it did for patients (*p* < 0.05). This indicated that patients performed significantly different depending on the start or stop event detection.

Regarding the WD value, ST obtained the best results for both start and stop models (bold text in Table 6), with lower FP/min and higher TPR and Acc. Looking at Table 7, WD results were similar in average for healthy subjects and patients, not showing the apparently superior performance that offline analysis attributed to healthy subjects. The same table also shows that ST presented the highest WD value for both healthy subjects and patients. HHT was again the method with the most irregular performance, as the lower WD value and higher standard deviation in Tables 6, 7 indicate. The HHT result was specially low in the case of the stop model of patients which was the reason of the previously detected difference in the pairwise comparison.

## 4. Discussion

A new BMI based on ST has been compared to another signal analysis technique (HHT) and a traditional transform (FFT). The tests were done for sixteen different subjects: eight healthy and eight with lower limb disabilities. With the help of a recently developed comprehensive index (WD), the different processing methods were evaluated in an offline and a pseudo-online scenario.

From the point of view of the differences between start and stop event detection of gait, offline analysis seemed to perform better for the stop detection. However, the pseudo-online approach offered a similar performance in average, with the same WD value in the case of the ST method. In addition, statistical analysis showed no significant differences between the start and stop models. Therefore, as pseudo-online model is a more adequate way to represent the performance of the BMI in a real-time scenario, it can be concluded that both event detection models (start/stop) are similar.

Another conclusion is related to the individual performance of the sixteen participants. Results of Tables 2, 5 show that BMI performance was dependent on the subject, as the performance of each of them was substantially different. This means that the subjects need some time to get used to the BMI in order to improve their results. However, there were not significant differences between healthy subjects and patients in the MANOVA test. Therefore, it is not needed to personalize the BMI depending on the type of subject.

Regarding the different methods of analysis, indices showed that ST obtained the best results with better Acc and TPR, and even zero FP/min for certain subjects. All the models showed higher WD value in average for ST (bold text in the tables) which demonstrates the better performance of this transform. This is mainly due to the analytical nature of ST that makes it a more robust method than HHT. HHT had an irregular performance with the lowest WD value for the start offline model of both type of subjects and the stop pseudo-online model of patients (WD < 0.4), but with similar results to the other methods in the rest of the cases (WD > 0.55). HHT was also the method with the highest standard deviation for all the models. The cause of this irregular behavior is the EMD algorithm. EMD did not always achieve to extract the different components related to the bands of frequency considered in the paper. If the EMD of an epoch mixes several tones in a IMF, *H*(ω, *t*) is not computed correctly and the *h*(ω) does not provide the three features per electrode in a constant way, which affects the classifier and the event detection. FFT performed as the second best method, but as it is not based on instantaneous amplitude and frequency, provided a worse determination of the transition from a relax to a starting gait state, and from a gait to a stopping gait state than ST.

The comparison of this work with previous works is not trivial. First, there are not many studies about detection of intention of start and stop gait for lower limb that provide the three parameters: TPR, FP/min and Acc. In addition, the FP/min can be computed differently depending on the approach. For instance, a different number of consecutive detections could be specified, or a statistical mode threshold could be used. And finally, WD is hardly used as a comparison index because it was recently developed.

In Jiang et al. (2015) an offline approach for single-trial detection of gait initiation from movement related cortical potentials was presented. The study, carried out for nine subjects, provided the following averaged results: TPR = 76.80 ± 8.97% and FP/min = 2.93 ± 1.09. No Acc was indicated in the paper, being TPR a bit lower and FP/min a bit higher than the offline ST results shown in Table 3 for the start model (TPR = 83.45 ± 10.98% and FP/min = 2.88 ± 1.27). Looking at previous work of the authors based on FFT (Hortal et al., 2016b), Table 2 of the reference shows averaged indices of TPR = 54.8 ± 9.3% and FP/min = 2.66 ± 2.24 for the offline start and stop gait intention of six subjects (no Acc provided). This example allows to compare the results in a similar scenario with more subjects under analysis. In our research, the same averaged indices (start and stop) show also an improvement: TPR = 78.82 ± 12.39% and FP/min = 2.25 ± 1.28.

It has been demonstrated that the BMI developed allows to detect the start and stop of gait intention through the use of EEG signals improving the accuracy obtained. Future research will aim the online implementation of the BMI with a motion assistant device. This approach could be useful in the context of the lower limb rehabilitation for patients that have suffered stroke.

## Author contributions

MO is responsible for the design, implementation and data analysis. MR-U and EI developed the classifier module and MO adapted it. Data acquisition was designed and performed by EI. MO, MR-U, EI and JA contributed to the revision process. JA actively contributed as director of the work.

### Conflict of interest statement

The authors declare that the research was conducted in the absence of any commercial or financial relationships that could be construed as a potential conflict of interest.

## References

[B1] Amzica F. Steriade M. (1998). Electrophysiological correlates of sleep delta waves. Electroencephalogr. Clin. Neurophysiol. 107, 69–83. 10.1016/S0013-4694(98)00051-0 9751278

[B2] Barrios L. J. Hornero R. Pérez-Turiel J. Pons J. L. Vidal J. Azorín J. M. (2017). State of the art in neurotechnologies for assistance and rehabilitation in spain: fundamental technologies. Rev. Iberoamer. Autom. Inf. Indust. 14, 346–354. 10.1016/j.riai.2017.06.003

[B3] Bracewell R. N. Bracewell R. N. (1986). The Fourier Transform and Its Applications. New York, NY: McGraw-Hill.

[B4] Cantero J. L. Atienza M. Stickgold R. Kahana M. J. Madsen J. R. Kocsis B. (2003). Sleep-dependent θ oscillations in the human hippocampus and neocortex. J. Neurosci. 23, 10897–10903. Available online at: http://www.jneurosci.org/content/23/34/10897?ct= 14645485 10.1523/JNEUROSCI.23-34-10897.2003 PMC6740994

[B5] Cheron G. Duvinage M. De Saedeleer C. Castermans T. Bengoetxea A. Petieau M.. (2012). From spinal central pattern generators to cortical network: integrated BCI for walking rehabilitation. Neural Plast. 2012:375148. 10.1155/2012/375148 22272380 PMC3261492

[B6] Cramer S. C. (2008). Repairing the human brain after stroke. II. restorative therapies. Ann. Neurol. 63, 549–560. 10.1002/ana.21412 18481291

[B7] Da Silva F. L. (2010). EEG: origin and measurement, in EEG-fMRI: Physiological Basis, Technique, and Applications, eds Mulert C. Lemieux L. (Berlin; Heidelberg: Springer), 19–38. 10.1007/978-3-540-87919-0_2

[B8] Field A. (2009). Discovering Statistics Using SPSS. London, UK: Sage Publications.

[B9] Gharabaghi A. (2016). What turns assistive into restorative brain-machine interfaces? Front. Neurosci. 10:456. 10.3389/fnins.2016.00456 27790085 PMC5061808

[B10] Ghasemi A. Zahediasl S. (2012). Normality tests for statistical analysis: a guide for non-statisticians. Int. J. Endocrinol. Metab. 10:486. 10.5812/ijem.3505 23843808 PMC3693611

[B11] Gwin J. T. Gramann K. Makeig S. Ferris D. P. (2011). Electrocortical activity is coupled to gait cycle phase during treadmill walking. Neuroimage 54, 1289–1296. 10.1016/j.neuroimage.2010.08.066 20832484

[B12] Hortal E. Planelles D. Iáñez E. Costa A. Úbeda A. Azorín J. M. (2016a). Detection of gait initiation through a ERD-based brain-computer interface, in Advances in Neurotechnology, Electronics and Informatics, eds Londral A. R. Encarnação P. (Cham: Springer), 141–150. 10.1007/978-3-319-26242-0_10

[B13] Hortal E. Úbeda A. Iáñez E. Azorín J. M. Fernández E. (2016b). EEG-based detection of starting and stopping during gait cycle. Int. J. Neural Syst. 26:1650029. 10.1142/S0129065716500295 27354191

[B14] Huang N. E. Shen Z. Long S. R. Wu M. C. Shih H. H. Zheng Q. (1998). The empirical mode decomposition and the Hilbert spectrum for nonlinear and non-stationary time series analysis. Proc. R. Soc. Lond. A Math. Phys. Eng. Sci. 454, 903–995. 10.1098/rspa.1998.0193

[B15] Jiang N. Gizzi L. Mrachacz-Kersting N. Dremstrup K. Farina D. (2015). A brain–computer interface for single-trial detection of gait initiation from movement related cortical potentials. Clin. Neurophysiol. 126, 154–159. 10.1016/j.clinph.2014.05.003 24910150

[B16] Li J. Ji H. Cao L. Zang D. Gu R. Xia B.. (2014). Evaluation and application of a hybrid brain computer interface for real wheelchair parallel control with multi-degree of freedom. Int. J. Neural Syst. 24:1450014. 10.1142/S0129065714500142 24694169

[B17] McFarland D. J. McCane L. M. David S. V. Wolpaw J. R. (1997). Spatial filter selection for EEG-based communication. Electroencephalogr. Clin. Neurophysiol. 103, 386–394. 10.1016/S0013-4694(97)00022-2 9305287

[B18] Ortiz M. Iáñez E. Rodriguez-Ugarte M. Azorín J. (2017). Empirical mode decomposition use in electroencephalography signal analysis for detection of starting and stopping intentions during gait cycle, in 26th IEEE International Symposium on Robot and Human Interactive Communications, (Lisbon: IEEE), 1–7.

[B19] Pfurtscheller G. Neuper C. (1994). Event-related synchronization of mu rhythm in the EEG over the cortical hand area in man. Neurosci. Lett. 174, 93–96. 10.1016/0304-3940(94)90127-9 7970165

[B20] Rao R. P. (2013). Brain-Computer Interfacing: An Introduction. Cambridge, UK: Cambridge University Press.

[B21] Rilling G. Flandrin P. (2006). On the influence of sampling on the empirical mode decomposition, in Acoustics, Speech and Signal Processing, 2006. ICASSP 2006 Proceedings. 2006 IEEE International Conference on, Vol. 3 (Toulouse: IEEE), III.

[B22] Rilling G. Flandrin P. (2008). One or two frequencies? The empirical mode decomposition answers. IEEE Trans. Signal Process. 56, 85–95. 10.1109/TSP.2007.906771

[B23] Rilling G. Flandrin P. Gonçalves P. (2003). On empirical mode decomposition and its algorithms, in IEEE-EURASIP Workshop on Nonlinear Signal and Image Processing, Vol. 3 (Grado: IEEER), 8–11.

[B24] Rodríguez-Ugarte M. Iáñez E. Ortíz M. Azorín J. M. (2017). Personalized offline and pseudo-online BCI models to detect pedaling intent. Front. Neuroinformatics 11:45. 10.3389/fninf.2017.00045 28744212 PMC5504298

[B25] Sburlea A. I. Montesano L. Minguez J. (2017). Advantages of EEG phase patterns for the detection of gait intention in healthy and stroke subjects. J. Neural Eng. 14:036004. 10.1088/1741-2552/aa5f2f 28291737

[B26] Severens M. Nienhuis B. Desain P. Duysens J. (2012). Feasibility of measuring event related desynchronization with electroencephalography during walking, in Engineering in Medicine and Biology Society (EMBC), 2012 Annual International Conference of the IEEE (San Diego, CA: IEEE), 2764–2767. 10.1109/EMBC.2012.6346537 23366498

[B27] Steinwart I. Christmann A. (2008). Support Vector Machines. New York, NY: Springer Science & Business Media.

[B28] Stockwell R. G. Mansinha L. Lowe R. (1996). Localization of the complex spectrum: the s transform. IEEE Trans. Signal Process. 44, 998–1001. 10.1109/78.492555

[B29] Subasi A. (2005). Automatic recognition of alertness level from EEG by using neural network and wavelet coefficients. Expert Syst. Appl. 28, 701–711. 10.1016/j.eswa.2004.12.027

[B30] Villarejo Mayor J. J. Costa R. M. Frizera-Neto A. Bastos T. F. (2017). Decoding of grasp and individuated finger movements based on low-density myoelectric signals. Rev. Iberoam. Autom. Infor. Indust. 14, 184–192. 10.1016/j.riai.2017.02.001

[B31] Wagner J. Solis-Escalante T. Grieshofer P. Neuper C. Müller-Putz G. Scherer R. (2012). Level of participation in robotic-assisted treadmill walking modulates midline sensorimotor EEG rhythms in able-bodied subjects. Neuroimage 63, 1203–1211. 10.1016/j.neuroimage.2012.08.019 22906791

